# Culture-Based Screening of Aerobic Microbiome in Diabetic Foot Subjects and Developing Non-healing Ulcers

**DOI:** 10.3389/fmicb.2016.01792

**Published:** 2016-11-22

**Authors:** Saba Noor, Jamal Ahmad, Iqbal Parwez, Maaz Ozair

**Affiliations:** ^1^Rajiv Gandhi Centre for Diabetes and Endocrinology, J.N Medical College, Aligarh Muslim UniversityAligarh, India; ^2^Department of Zoology, Faculty of Life Sciences, Aligarh Muslim UniversityAligarh, India

**Keywords:** Type 2 diabetes, Diabetic foot infection, Multi-drug resistant organisms, Non-healing ulcers, Antibiogram

## Abstract

The study was carried on diabetic foot patients to deduce clinical attributes, the occurrence of the range of aerobic microbial flora and to assess their comparative *in vitro* susceptibility to the customarily used antimicrobials. We also studied the potential risk factors involved in the development of non-healing ulcers. A total of 87 organisms were isolated from 70 specimens, including *Escherichia coli* (19.5%) among the Gram-negative and *Staphylococcus aureus* (18.4%) among the Gram-positive as the predominant aerobes explored. *Pseudomonas aeruginosa* and *E. coli* were predominant isolates of non-healing ulcers. The antimicrobial sensitivity pattern revealed that vancomycin (100%) and amikacin (90.4%) exhibited highest sensitivity to Gram-positive cocci, while all strains of *P. aeruginosa* were sensitive toward imipenem (100%). The prevalent uncontrolled glycemic status, altered lipid spectra, the existence of neuropathy, and peripheral vascular disease, suggested predisposition toward the development of non-healing lesions. The study has underlined the need for continuous surveillance of bacteria and their antimicrobial sensitivity blueprints to provide the basis for empirical therapy and to minimize the risk of complications. Further, stringent clinical evaluation, and medical history will help in revealing the risk of developing non-healing status in diabetic foot ulcers.

## Introduction

Diabetes mellitus being the global epidemic of 21^st^ century, ultimately leads to end-organ damage due to hyperglycemia, imposing a major health burden. Diabetes-associated foot ulcers, followed by infection causes substantial morbidity and dreaded complications like systemic toxicity, gangrene, and lower extremity loss. The cumulative lifetime incidence for the development of foot ulcers in diabetes is as high as 25% (Noor et al., 2015). More than 85% of lower extremity amputations in patients with diabetes are preceded by foot ulcers (Pecoraro et al., 1990). Diabetic peripheral neuropathy and peripheral arterial disease are the key etiologic agents in foot ulceration. These may act together with other factors such as biomechanical, immunological imbalances, microvascular disease, foot deformities, hindered joint mobility, and increased susceptibility to infection thereby representing diabetic foot ulcers (DFUs) as a major social, medical and economic problem of developing countries (Eneroth et al., 1999). Infection is diagnosed on the presence of ≥2 classical signs of inflammation (erythema, edema, and purulence). Spanning the spectrum from simple, superficial cellulitis, microbial flora can lead to chronic osteomyelitis, and dreaded systemic toxicity along with gangrenous limbs resulting in lower extremity amputation (Lipsky et al., 2004). Often in association with lack of sensation, stiffened arteries, compromised host immune response, and recurrence of ulcers especially harbored by multidrug-resistant organisms places diabetic foot patients at higher risk of non-healing ulcers. Earlier studies have shown that most diabetic foot infections are polymicrobial with a predominance of Gram-positive cocci especially *S. aureus*, and *Streptococci* (Sapico et al., 1984; Jones et al., 1985). However, recent studies indicate the dominance of Gram-negative pathogens in the monomicrobial state, particularly members of family *Enterobacteriace* and *Pseudomonas* (Tiwari et al., 2011; Turhan et al., 2013). The prolonged course of antimicrobials for uninfected lesion or healed wounds, long hospital stay, and surgical measures may predispose patients to colonization and infection with drug-resistant pathogens and associated adverse outcomes. Multidrug-resistant organisms like methicillin-resistant *Staphylococcus aureus* (MRSA), metallo-beta-lactamases (MBL), extended-spectrum beta-lactamases (ESBL) producers, and ampicillin-resistant beta-lactamases (AmpC) producers further complicate the aura making lower extremity loss more common (Dang et al., 2003; Tascini et al., 2006; Kandemir et al., 2007; Richard et al., 2008). Proper wound management requires early analysis of infection and prompt initiation of appropriate antimicrobial therapy.

For infected ulcers, a post-debridement specimen from tissue should be obtained and processed for detection of causative pathogens (Zubair et al., 2010). Antibiotic susceptibility testing is a prerequisite for the management of infections which can help to make better therapeutic choices.

The present study was designed to characterize common bacterial microbes in diabetic foot wounds, explore the drug sensitivity pattern of isolates and analysis of changing etiology of DFUs in North Indian population. Since poor healing status is a major cause of lower extremity amputations in diabetics, the study is also aimed to uncover the potential risk factors associated with the development of non-healing ulcers in diabetics.

## Research designs and methods

### Study designs

A prospective hospital-based study was conducted in Jawaharlal Nehru Medical College, Aligarh Muslim University, Aligarh, India during the period from July 2014 to March 2016. Ninety type 2 diabetic patients with foot ulcers were admitted to endocrinology ward. Seventy patients with infected DFUs were enrolled in the study. All the subjects gave informed consent, and clearance was obtained from the Bio-Ethical Committee (BEC), Faculty of Medicine, J.N. Medical College, Aligarh Muslim University, Aligarh.

### Clinical examination

A well-structured questionnaire was developed for a detailed history and physical examination. Subjects were clinically assessed for age, sex, body mass index (BMI), lipid profile, duration of diabetes, glycemic status, liver, and renal functionality. Patients were also clinically evaluated for presence of other comorbidities such as retinopathy (fundoscopy), nephropathy (creatinine >1.5 mg% or presence of micro- or macro-albuminuria), neuropathy (absence of perception of the Semmes—Weinstein monofilament at 2 of 10 standard planter sites on either foot), peripheral vascular disease (ischemic symptoms and intermittent claudication of rest pain, with or without absence of pedal pulses or posterior tibial pulses) and hypertension (previous medication of anti-hypertensive drug or a BP ≥ 140/90 mmHg). Clinical assessment of infection in the wound was made with the occurence of classic signs of inflammation (redness, swelling, tenderness, warmth, or pain) or purulent secretions or additional minor signs of non-purulent secretions (friable or discolored granulation tissue, undermining of wound edges, foul odor). Duration and size of ulcer were calculated by multiplying longest and widest diameters and expressed in centimeter square. Ulcers were graded using Wagner classification system, grade I (superficial ulcer or ulcer of subcutaneous tissue), grade II (ulcers extended into tendon, bone, or capsule), grade III (deep ulcer with osteomyelitis, or abscess), and grade IV (gangrene of toes). Subjects with grade 0 (uninfected lesions/ intact skin/ healed ulcers) were debarred from the study. Amputation was defined as the complete loss in the transverse anatomical plane of any part of the lower limb. Diagnosis of infection involving bone was done by either using a sterile probe in exposed bone or evidence obtained from plain radiographs or magnetic resonance imaging (MRI) in the absence of sinus tract.

### Specimen collection

Pus aspirates or soft tissue samples were collected on the day of admission after proper cleaning of the diabetic wound with saline followed by debridement of superficial tissue exudates and promptly sent to a laboratory and processed for aerobic bacterial identification as described by Gadepalli et al. (2006).

Venous blood samples were collected after an overnight fast and centrifuged at 3000 rpm for 5 min at 4°C. Serum or plasma was immediately separated and stored in aliquots at −80°C until further analysis.

### Microbiological analysis

All pus samples were Gram-stained and the bacteria were isolated by inoculation of specimens on a set of selective and non-selective media such as blood agar, MacConkey agar, and nutrient agar (Hi Media, Mumbai, India), and were incubated at 37°C overnight. Isolated organisms were identified on the basis of culture characteristics, colony morphology, and biochemical reactions as per the standard protocols (Collee et al., 1996).

### Susceptibility testing

Antimicrobial susceptibility testing of aerobic isolates was done by using Mueller-Hinton agar using Kirby-Bauer disk diffusion method as recommended by Clinical and Laboratory Standards Institute (2006b). The antibiotic panels for each group of isolates were selected according to the Clinical and Laboratory Standards Institute (2006a). Antimicrobials used in the study were, cefotaxime (30 μg), cefepime (30 μg), imipenem (10 μg), cefixime (5 μg), cefoperazone (75 μg), cefoperazone/sulbactam (75/10 μg), ceftazidime (30 μg), amikacin (30 μg), ceftriaxone (30 μg), oxacillin (1 μg), piperacillin (100 μg), piperacillin/tazobactam (100/10 μg), cefoxitin (30 μg), chloramphenicol (30 μg), gentamicin (10 μg), levofloxacin (5 μg), sparfloxacin (5 μg), streptomycin (10 μg), vancomycin (30 μg), tobramycin (10 μg), and erythromycin (15 μg). Dried disks were stored in refrigerator. An appropriate dilution of a broth culture was spread on Mueller-Hinton agar plate using sterile swabs. Plates were dried at 37°C for 30 min, and antibiotic disks (4 or 5 per 10 cm plate) were applied with sterile forceps. After overnight incubation at 37°C, the level of sensitivity was determined by measuring the zones of inhibition of growth around the disks. Growth was inhibited around disks containing antimicrobials to which the bacterium was susceptible but not around those to which it was resistant.

### Clinical investigations

Plasma/ serum obtained at the day of admission were processed for estimation of HbA1c by ion- exchange high-performance liquid chromatography (Bio-Rad D-10, India) and glucose estimation by colorimetric assays. Serum lipid analysis (triglycerides, total cholesterol, LDL- cholesterol, HDL-cholesterol, VLDL-cholesterol, and phospholipids) was done using commercially available kits (Avantor, U.S.A) according to manufacturer guidelines (Allain et al., 1974).

### Statistical analysis

Quantitative variables were represented as means ± standard deviation and categorical data as a percentage (%). Student's *t*-test or chi- square test was used to compare the differences between the groups. A two-tailed *p* < 0.05 was considered as statistically significant. Statistical analyses were carried out using Medcalc software version 15.11.4.

## Results

### Demographical variables

Out of 90 type 2 diabetic patients with DFUs admitted to endocrinology ward, seventy had infected DFUs. Patients were grouped depending upon healing and non-healing state of ulcer following routine examination based on healing signs/symptoms. Routine clinical examination of ulcers was done up-to 2 months from the day of admission to evaluate the healing status. Non- healing lesions were clinically diagnosed on the basis of absence of epithelialization, angiogenesis, and formation of granulation tissues. Patients with non-healing wounds were significantly older than those without it (*p* < 0.05). HbA1c (*p* < 0.05), fasting plasma glucose (*p* < 0.05), serum triglyceride (*p* < 0.01), and low density lipoprotein (*p* < 0.01) were significantly elevated in subjects with non-healing ulcers as compared to those without it. Patients with non- healing infected ulcers had had a higher prevalence of neuropathy (*p* < 0.05), peripheral vascular disease (*p* < 0.05), and osteomyelitis (*p* < 0.05) than those with healing ulcers. Surgical amputations of lower limb were significantly higher in subjects with non-healing wounds (*p* < 0.05; Table 1).

**Table 1:** **Clinical and demographical variables of subjects with healing and non-healing ulcers**.

**Characteristics**	**Healing Ulcers**	**Non-healing Ulcers**	***p* value**
N	27	43	
Male (%)	22 (78.5%)	29 (82.5%)	NS
Age (yrs)	50.2±10.8	55.9±11.6	<**0.05**
Diabetes duration (yrs)	7.6±5.5	7.4±5.1	NS
BMI (kg/m^2^)	25.5±5.2	31.1±3.1	<**0.0001**
HbA1c (%)	9.1±2.0	11±3.6	<**0.05**
FPG (mg/dL)	170±44.2	200.7±58.6	<**0.05**
Se. Cr (mg/dL)	1.4±0.8	1.2±0.5	NS
**LIPID PROFILE**			
TC (mg/dL)	164.2±22.4	170.2±29.43	NS
TAGs (mg/dL)	148.3±38.9	183.1±54.8	<**0.01**
HDL (mg/dL)	47.6±10.1	39.4±6.6	<**0.0001**
LDL (mg/dL)	71.6±11.3	78.0±6.1	<**0.01**
**SIZE OF ULCER (cm**^2^**)**			
<4 cm^2^	13 (48.1%)	12 (27.9%)	<**0.05**
≥4 cm^2^	14 (51.8%)	31 (72%)	
Ulcer duration (months)	1.2±0.7	1.7±0.6	<**0.05**
**GRADE OF ULCER**				
1	1 (3.7%)	2 (4.6%)	NS
2	11 (40.7%)	13 (30.2%)	NS
3	5 (18.5%)	12 (27.9%)	NS
4	8 (29.6%)	16 (37.2%)	NS
**COMPLICATIONS**			
Hypertension	11 (39.2%)	18 (45.7%)	NS
Retinopathy	20 (71.4%)	29 (77.1%)	NS
Neuropathy	16 (39%)	12 (24.3%)	<**0.05**
Nephropathy	24 (85.3%)	32 (95.4%)	NS
Peripheral vascular disease	15 (53.5%)	29 (78.3%)	<**0.05**
Osteomyelitis	12 (35.7%)	31 (78.3%)	<**0.05**
**Amputation**	8 (29.6%)	25 (58.1%)	<**0.05**
Minor	8 (29.6%)	22 (51.1%)	NS
Major	0 (0%)	3 (6.9%)	

*N, number of subjects; BMI, body mass index; HbA1c, glycated hemoglobin; FPG, fasting plasma glucose; Se. Cr, serum creatinine; TC, total cholesterol; TAG, triglycerides; HDL, high density lipoprotein; LDL, low density lipoprotein; NS, not significant*.

*The bold values denote the p value obtained after applying student t-test and Chi- square statistical analysis when when variables of healing ulcer group were compared with non-healing ulcer group*.

### Ulcer characteristics

Among the subjects, the mean duration of infected ulcer was 1.45 ± 0.6 months. Size of ulcer ≥4 cm^2^ was observed in 45 (64.3%) cases and <4 cm^2^ in 25 (35.7%) subjects. Surgical amputation was done in 33 (47.1%) subjects, of which 3 (4.3%) underwent major amputation, and 30 (42.85%) were subjected to minor amputation. Diabetic foot wounds of non-healing category were significantly long-standing than healing wounds (*p* < 0.05) (Table 1). Of total 70 subjects, 19 (27.1%) were on antibiotics before admission, 10 (14.3%) were unaware of previous medication whereas rest of the subjects did not received antibiotics. Seventy percent patients had no prior knowledge of diabetes linked secondary complications and foot care.

### Wound microbiology

A total of 87 aerobic isolates were identified from 70 ulcer specimens, averaging 1.2 species per lesion. Fifty-three (75.7%) had monomicrobial infection, and polymicrobial etiology was observed in 17 (24.3%) cases. In our study Gram-negative bacilli were predominant (75.9%) than Gram-positive cocci (24.1%). In terms of relative abundance, *E. coli* accounted for (19.5%), followed by *S. aureus* (18.4%), *P. aeruginosa* (17.2%), *Klebsiella species* (14.9%), *Citrobacter species* (12.6%), *Proteus species* (11.5%), and *Enterococcus faecialis* (5.7%).

Subjects with non-healing ulcers during hospital stay had a predominance of *E. coli* and *P. aeruginosa* (13.8% each), followed by *S. aureus* (12.6%). *Klebsiella species* (10.3%), followed by *Citrobacter species* (9.2%) were most frequent in healing wounds (Table 2).

**Table 2:** **Distribution of 87 isolates in healing and non-healing diabetic foot ulcers**.

**Name of isolates**	**Healing ulcers**	**Non-healing ulcers**
	***n* (%)**	***n* (%)**
*Escherichia coli*	5 (5.7%)	12 (13.8%)
*Staphylococcus aureus*	5 (5.7%)	11 (12.6%)
*Pseudomonas aeruginosa*	3 (3.4%)	12 (13.8%)
*Klebsiella species*	9 (10.3%)	4 (4.6%)
*Citrobacter species*	8 (9.2%)	3 (3.4%)
*Proteus species*	5 (5.7%)	5 (5.7%)
*Enterococcus faecialis*	2 (2.3%)	3 (3.4%)

*n, number of isolates*.

Based on antibiotic susceptibility pattern, 62.5% strains of *S. aureus* were found to be resistant to levofloxacin. All strains of Gram-positive cocci showed sensitivity toward vancomycin (100%), amikacin (90.5%), and ciprofloxacin (71.4%). MRSA accounted for 25% of isolated strains of *S. aureus* and were resistant to oxacillin (Table 3).

**Table 3:** **Antibiogram of Gram-positive cocci**.

	***Staphylococcus aureus* (*n* = 16)**		***Enterococcus faecialis* (*n* = 5)**		**Total (*n* = 21)**	
**Antibiotics**	**S (%)**	**R (%)**	**S (%)**	**R (%)**	**S (%)**	**R (%)**
Amikacin	14 (87.5%)	2 (12.5%)	5 (100%)	0 (0%)	19 (90.5%)	2 (9.5%)
Erythromycin	10 (62.5%)	6 (37.5%)	4 (80%)	1 (20%)	14 (66.7%)	7 (33.3%)
Ciprofloxacin	11 (68.75%)	5 (31.25%)	4 (80%)	1 (20%)	15 (71.4%)	6 (28.6%)
Gentamicin	10 (62.5%)	6 (37.5%)	4 (80%)	1 (20%)	14 (66.7%)	7 (33.3%)
Levofloxacin	6 (37.5%)	10 (62.5%)	4 (80%)	1 (20%)	10 (47.6%)	11 (52.4%)
Oxacillin	11 (68.75%)	5 (31.25%)	5 (100%)	0 (0%)	16 (76.2%)	5 (23.8%)
Vancomycin	16 (100%)	0 (0%)	5 (100%)	0 (0%)	21 (100%)	0 (0%)

*S, sensitive; R, resistant; n, number of isolates*.

In *E. coli*, the majority of strains were resistant to cefoperazone (82.35%), followed by gentamicin, levofloxacin, and tobramycin (70.6% each). *Klebsiella species* were found to be highly resistant to cefixime (84.6%) and cefoperazone (69.2%). *Citrobacter species* showed highest resistance to cefoperazone (81.8%) and ceftriaxone (81.8%), while resistance to gentamicin and cefepime was 63.6% each. High level of resistance to amikacin, cefoperazone, and tobramycin (70% each) was found in *Proteus species* (Table 4).

**Table 4:** **Antibiogram of Enterobacteriace**.

**Antibiotics**	***Escherichia coli* (*n* = 17)**		***Klebsiella species* (*n* = 13)**		***Citrobacter species* (*n* = 11)**		***Proteus species* (*n* = 10)**		**Total (*n* = 51)**	
	**S (%)**	**R (%)**	**S (%)**	**R (%)**	**S (%)**	**R (%)**	**S (%)**	**R (%)**	**S (%)**	**R (%)**
Amikacin	13 (76.5%)	4 (23.5%)	9 (69.2%)	4 (30.8%)	5 (45.4%)	6 (54.5%)	3 (30%)	7 (70%)	30 (58.8%)	21 (41.2%)
Cefoperazone	3 (17.6%)	14 (82.35%)	4 (30.8%)	9 (69.2%)	2 (18.2%)	9 (81.8%)	3 (30%)	7 (70%)	15 (29.4%)	22 (43.1%)
Cefixime	6 (35.3%)	11 (64.7%)	2 (15.4%)	11 (84.6%)	10 (90.9%)	1 (9.0%)	6 (60%)	4 (40%)	24 (47.0%)	27 (52.9%)
Cefepime	6 (35.3%)	11 (64.7%)	10 (76.9%)	3 (23.1%)	4 (36.4%)	7 (63.6%)	5 (50%)	5 (50%)	25 (49.0%)	26 (51.0%)
Cefetriaxone	6 (35.3%)	11 (64.7%)	10 (76.9%)	3 (23.1%)	2 (18.2%)	9 (81.8%)	4 (40%)	6 (60%)	22 (43.1%)	29 (56.9%)
Gentamicin	5 (29.4%)	12 (70.6%)	6 (46.15%)	7 (53.8%)	4 (36.4%)	7 (63.6%)	4 (40%)	6 (60%)	19 (37.2%)	32 (62.7%)
Levofloxacin	5 (29.4%)	12 (70.6%)	10 (76.9%)	3 (23.1%)	6 (54.5%)	5 (45.4%)	4 (40%)	6 (60%)	25 (49.0%)	26 (51.0%)
Tobramycin	5 (29.4%)	12 (70.6%)	6 (46.15%)	7 (53.8%)	6 (54.5%)	5 (45.4%)	3 (30%)	7 (70%)	20 (39.2%)	30 (58.8%)
Cefeperazone + Sulbactam	8 (47.0%)	9 (52.9%)	12 (92.3%)	1 (7.7%)	5 (45.4%)	6 (54.5%)	6 (60%)	4 (40%)	31 (60.8%)	20 (39.2%)

*S, sensitive; R, resistant; n, number of isolates*.

Among the isolates of *P. aeruginosa*, piperacillin (53.3%), and piperacillin + tazobactam (86.6%) showed good activity. All strains of *Pseudomonas aeruginosa* were found to be sensitive to imipenem (Table 5).

**Table 5:** **Antibiogram of *Pseudomonas aeruginosa***.

	***Pseudomonas aeruginosa (n* = 15)**	
**Antibiotics**	**S (%)**	**R (%)**
Amikacin	5 (33.3%)	10 (66.7%)
Ceftazidime	4 (26.7%)	11 (73.3%)
Cefepime	4 (26.7%)	11 (73.3%)
Levofloxacin	6 (40.0%)	9 (60.0%)
Sparfloxacin	4 (26.7%)	11 (73.3%)
Tobramycin	5 (33.3%)	10 (66.7%)
Piperacillin	8 (53.3%)	7 (46.7%)
Piperacillin + Tazobactam	12 (80.0%)	3 (20.0%)
Imipenem	15 (100%)	0 (0%)

*S, sensitive; R, resistant; n, number of isolates*.

## Discussion

This study presents a comprehensive clinical and microbiological contour of infected DFUs in hospitalized patients. Patients with non-healing ulcers were older and had poor glycemic status than those with healing wounds. Impaired insulin secretion/action causes a halt in the uptake of glucose, leading to reduced activity of fibroblasts, and polymorphonuclear neutrophils causing poor healing (Rosenberg, 1990).

Subjects with non-healing lesions had a high prevalence of neuropathy and peripheral vascular disease. Loss of sensation of peripheral arteries leads to recurrent ulcers with susceptibility to develop multidrug-resistance isolates due to heavy prior treatment with antimicrobials, thus causing delayed healing. Inadequate blood flow caused due to thickening of arteries of lower extremities creates an ischemic environment and compromised mobility causing prolonged periods of unrelieved pressures on the extremities. This results in increased shearing force applied to the skin and underlying tissues leading to decrease in oxygen tension and eventual tissue necrosis (Defloor, 1999).

In the present study we observed that out of 87 aerobic bacteria isolated from 70 pus samples, an average of 1.2 organisms was found. These findings are similar to those reported by Viswanathan et al. (2002), where cultures yielded an average of 1.2 isolates per lesion, and were lower than the previous studies of Zubair et al. (2011), Gadepalli et al. (2006), and Shahid et al. (2009), with the reported average rate of isolation between 1.57, 2.3, and 5.8 respectively. The decrease in the number of isolates (1.2 organisms per lesion) in the present study in comparison to previously cited reports, could be because of widespread use of broad-spectrum antibiotics inclusive of piperacillin/tazobactam by the Primary Health Care physicians without obtaining proper pus culture before initiating antibiotic. Unlike Western countries reporting Gram-positive aerobes as predominant pathogens (Wang et al., 2010; Roberts and Simon, 2012), there has been a changing trend in the microorganisms causing DFI, with Gram-negative bacteria replacing Gram-positive bacteria in India (Ramakant et al., 2011; Umadevi et al., 2011). Interestingly, we have also observed the predominance of Gram-negative organisms in DFUs in our study and the same has also been reported by Mehta et al. (2014) and Shankar et al. (2005). However, previous studies reported the dominance of Gram-positive aerobes in diabetic foot infection (Mantey et al., 2000; Fejfarová et al., 2002). The ratio of Gram-negative to Gram-positive aerobes in our study was 2.9, which is lower than reported by Gadepalli et al. (2006). The prevalence of this kind of discrepancy in findings, could be because of geographical variations, age-sex composition, grading of ulcers and study settings included in the analysis.

In the present study, *E. coli* (19.5%), and *S. aureus* (18.3%) were the most predominating Gram-negative and Gram-positive isolates respectively. These findings are similar to those reported by Kandati et al. (2015) and Girish and Kumar (2011), from Southern India. Among non- healing ulcers, *E. coli* and *P. aeruginosa* (13.7% each), were most prevalent aerobes. This raises a serious concern for aggressive patterns of Gram-negative aerobes and their role in the poor healing of diabetic foot wounds.

All strains of *S. aureus* including MRSA were sensitive to vancomycin, which is similar to the findings of Reghu et al. (2016), Kandati et al. (2015), and Mehta et al. (2014), but against the findings of Girish and Kumar (2011).

Our study has shown that amongst the Enterobacteriace group, amikacin showed good antimicrobial activity against *E. coli*, followed by *Klebsiella species, Citrobacter species*, and *Proteus species*. Amikacin can, therefore, be considered as a better choice of drug in infection caused by these organisms. Our findings on the sensitivity of amikacin for Enterobacteriace are in tune with Girish and Kumar (2011), who reported the highest sensitivity of amikacin to *Citrobacter species* (100%), followed by *Proteus species* (99%), *E. coli* (86%) and *Klebsiella species* (72%). Our study also indicates that *Pseudomonas* infection can respond better to imipenem, which is corroborated by the earlier reports quoting imipenem, the best sensitive drug to *P. aeruginosa* (Mantey et al., 2000; Bansal et al., 2008).

Based on our result and what is documented in literature so far, it is amply clear that there is no antibiotic which can cover all isolates, and therefore, a combination of drugs has to be recommended to overcome the extensive multidrug-resistance. The emergence of resistant strains in DFIs is a major hurdle to our efforts to prevent limb loss as the infection is most common cause complicating the diabetic foot pathogenesis. Even if the pathogen is susceptible to one particular antimicrobial, the drug is unlikely to accomplish therapeutic concentration at the site of infection due to hindrance caused by deranged host immune responses, virulence factors, such as proteases, hemolysins, and collagenases that cause inflammation and impede wound healing contribute to the chronicity of the infection (Bowler and Davies, 1999; Von Eiff et al., 2002).

It must be considered that the present study is a prospective study but with highly significant results. A more comprehensive multi-centric study covering a diversity of population along with previous hospitalization details and prior antibiotic exposure is warranted to cover a wide array of microbial range. This information will form a substantive baseline data to elucidate the causes of high prevalence of non-healing ulcers amongst the Indian population.

## Conclusions

This study demonstrates the existence of a diversity of organisms in DFUs. The emerging dominance of Gram-negative aerobes replacing Gram-positive bacteria in infected diabetic foot wounds may impose a serious health burden in healing of ulcers. Therefore, antimicrobial prescribed should be broadened covering both Gram-negative and Gram-positive pathogens to improve the healing status in diabetic foot. Antimicrobial prescribed should also target multidrug-resistant strains which are a compounding trouble in treatment of diabetic foot infection and associated complications. In addition, a proper clinical analysis, including the presence of neuropathy and vascular disease, history of ulcerations should be routinely done in diabetics.

## Author contributions

SN performed experimental work along with statistical calculations. JA and IP conceived the hypothesis provided the overview during experimental work and helped in preparation of this manuscript into the final form. MO was involved in standardization of clinical methodologies and manuscript drafting.

### Conflict of interest statement

The authors declare that the research was conducted in the absence of any commercial or financial relationships that could be construed as a potential conflict of interest.
